# AI for the hemodynamic assessment of critically ill and surgical patients: focus on clinical applications

**DOI:** 10.1186/s13613-025-01448-w

**Published:** 2025-02-24

**Authors:** Frederic Michard, Marijn P. Mulder, Filipe Gonzalez, Filippo Sanfilippo

**Affiliations:** 1 MiCo, Vallamand, Switzerland; 2 Cardiovascular and Respiratory Physiology, University of Twente, Enschede, The Netherlands; 3 Centro Cardiovascular da Universidade de Lisboa, CCUL@RISE, Faculdade de Medicina, Universidade de Lisboa, Lisbon, Portugal; 4 Intensive Care Department of Hospital Garcia de Orta, Almada, Portugal; 5 Department of Surgery and Medical-Surgical Specialties, Section of Anesthesia and Intensive Care, University of Catania, Catania, Italy

**Keywords:** Artificial intelligence, Machine learning, Predictive analytics, Blood pressure, Cardiac output, Echocardiography

## Abstract

Several artificial intelligence (AI)-driven tools have emerged for the hemodynamic evaluation of critically ill and surgical patients. This article provides an overview of current developments and potential clinical applications of machine learning (ML) for blood pressure measurements, hypotension prediction, hemodynamic profiling, and echocardiography. ML algorithms have shown promise in enabling continuous, non-invasive blood pressure monitoring by analyzing pulse oximetry waveforms, though these methods require periodic calibration with traditional oscillometric brachial cuffs. Additionally, a variety of ML models have been trained to forecast impending hypotension. However, clinical research indicates that these algorithms often primarily rely on mean arterial pressure, leading to questions about their added predictive value. The issue of false-positive alerts is also significant and can result in unwarranted clinical interventions. In terms of hemodynamic profiling, ML algorithms have been proposed to automatically classify patients into specific hemodynamic endotypes. However, current evidence suggests these models tend to replicate conventional hemodynamic profiles found in medical textbooks or depicted on advanced hemodynamic monitors. This raises questions about their practical clinical utility, especially given occasional discrepancies that could impact treatment decisions. Point-of-care ultrasound (POCUS) has gained traction for evaluating cardiac function in patients experiencing circulatory shock. ML algorithms now embedded in some POCUS systems can assist by recognizing ultrasound images, guiding users for optimal imaging, automating and reducing the variability of key echocardiographic measurements. These capabilities are especially beneficial for novice operators, potentially enhancing accuracy and confidence in clinical decision-making. In conclusion, while several AI-based technologies show promise for refining hemodynamic assessment in both critically ill and surgical patients, their clinical value varies. Comprehensive validation studies and real-world testing are essential to identify which innovations will genuinely contribute to improving the quality of care.

## Background

Artificial intelligence (AI) refers to the development of systems or machines that mimic human intelligence to perform tasks such as learning, reasoning, problem-solving, and decision-making. Machine learning (ML) algorithms are a subset of AI that enable computers to learn patterns and make predictions or decisions without explicit programming by analyzing data and improving their performance over time. ML algorithms are increasingly used in medicine [1], and critical care is no exception [2, 3]. Contemporary computer power and ML algorithms offer the opportunity to analyze big data, including physiologic waveforms, at the bedside and in a fraction of a second. Predictive analytics, or the science of prediction, enables forecasting adverse events, such as hemodynamic instability, opening the door to proactive interventions [4, 5]. This could lead to more accurate, timely, and personalized patient care. Ultimately, AI solutions could decrease the risk of complications, improve patient outcomes, and reduce healthcare costs [1, 2]. However, hospital adoption requires ongoing validation through clinical studies demonstrating the superiority of AI-enabled solutions over human skills and existing tools and the development of user-friendly solutions.

Several AI-enabled tools have been developed for the hemodynamic assessment of critically ill and surgical patients. This article provides an overview of current developments and potential clinical applications of ML innovations for blood pressure (BP) measurements, hypotension prediction, hemodynamic profiling, and echocardiography.

## AI for blood pressure measurements

The oscillometric brachial cuff method is used to measure BP in most critically ill and surgical patients. It has the disadvantage of providing only intermittent measurements, and it is known to overestimate low BPs (and underestimate high BPs) compared to reference intra-arterial measurements [6]. Therefore, it does not permit the immediate detection of hypotensive events, which are linked with morbidity and mortality. In high-risk surgical and ICU patients suffering from circulatory shock, the use of intra-arterial monitoring is recommended but is associated with infectious, hemorrhagic, and thrombotic complications. Volume clamp techniques enable continuous and non-invasive BP monitoring with servo-controlled finger cuffs incorporating photoplethysmographic (PPG) sensors. Unfortunately, their accuracy and precision are influenced by peripheral circulation (typically shut down in patients with shock) [7], and a recent meta-analysis concluded that only one-third of validation studies reported that volume clamp techniques meet current international standards (bias < 5 mmHg and SD < 8 mmHg) for BP monitoring [8]. In addition, a meta-analysis of 7 randomized controlled trials suggested that using volume clamp methods during non-cardiac surgery does not improve postoperative patient outcomes [9]. Interestingly, thanks to ML algorithms, attempts have recently been made to compute BP directly from PPG waveforms. Such PPG waveforms can be recorded with pulse oximeters in all critically ill and surgical patients [10]. Computing BP from PPG waveforms would open the door to easy, non-invasive, and continuous BP monitoring in clinical situations where BP may quickly and significantly vary, from spinal anesthesia during cesarian section to general anesthesia induction, hemorrhage during and after surgery, and sepsis and cardiac complications in critically ill patients (Table 1).

**Table 1:** Machine learning algorithms for the hemodynamic assessment of critically ill and surgical patients

Designed for	Data input	Expected benefit	Comment
AP monitoring from pulse oximeter	Photoplethysmographic waveform	Easy non-invasive and continuous BP monitoring	Not commercially available
Detection of overdamping of the AP waveform	AP waveform	Quality indicator for AP measurements and derived variables (e.g. CO)	Not commercially available
Prediction of hypotension (MAP < 65 mmHg)	AP waveform	Proactive intervention to decrease the duration and depth of hypotensive events	• Commercially available • Does not predict hypotension better than MAP • High rate of false positives → risk of unjustified treatment
Hemodynamic profiling	• Ultrasound images • NT-ProBNP • Hemodynamic variables including CO	• Automatic identification of hemodynamic profiles • Better personalization of care	• Not commercially available • Superiority over existing methods not yet established
Automation of echocardiographic measurements (LVEF, VTI, IVC diameter)	Ultrasound images	• Guiding users for optimal imaging • Automating and reducing the variability of measurements	Commercially available

*AP* arterial pressure, *CO* cardiac output, *IVC* inferior vena cava, *LVEF* left ventricular ejection fraction, *MAP* mean arterial pressure, *VTI* velocity time integral

**Fig. 1:**
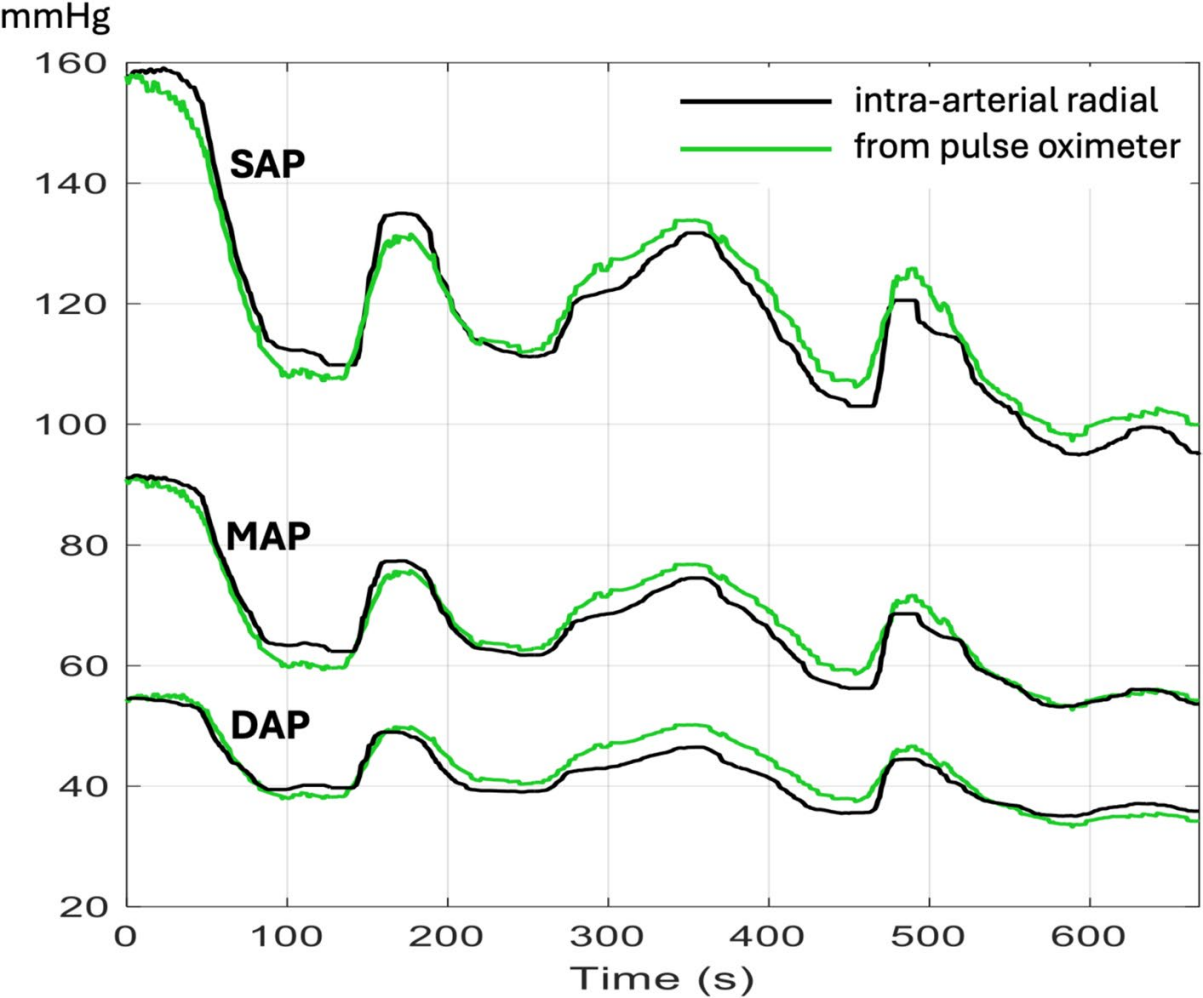
Example of continuous monitoring of systolic (SAP), mean (MAP), and diastolic (DAP) arterial pressure with a pulse oximetry signal

Machine learning algorithms can be trained with a large number of PPG waveforms and corresponding BP values. Thus, they can learn the specific PPG patterns associated with a drop or a rise in BP. Pending regular calibrations with an external technique (usually the oscillometric brachial cuff method), these algorithms allow the continuous estimation of BP from any PPG waveform. Several wristbands integrating optical sensors to record reflective PPG waveforms have been approved for medical use, although validation studies have reported conflicting findings [11, 12]. As of today, these wristbands are mainly proposed to track changes in BP in ambulatory patients with chronic hypertension. Nevertheless, the same principle could be applied to upgrade pulse oximeters used in surgical and critically ill patients. For instance, in patients undergoing surgery, Ghamri et al. [13] used the PPG signal from standard pulse oximeters and an ML algorithm to track changes in BP during anesthesia induction (Fig. 1). They reported a strong correlation between changes in systolic and mean BP measured by a radial arterial catheter (the reference method in this study) and those predicted by the PPG signal. Because oscillometric brachial cuffs and pulse oximeters are ubiquitous devices, one may easily envision a future where they will communicate, pulse oximeters enabling the continuous monitoring of BP between intermittent oscillometric measurements and triggering brachial cuff inflation anytime a significant change in BP is suspected [14]. Several studies suggest that, in addition to systolic, diastolic, and mean BP measurements, PPG waveforms coupled with continuous ECG recordings could be used to impute or rebuild BP waveforms [15, 16]. Whether these artificial BP waveforms could be useful to compute additional hemodynamic variables such as pulse pressure variation, stroke volume, and cardiac output remains to be determined. In addition, poor peripheral perfusion is a well-documented factor that compromises the accuracy of oxygen saturation measurements via pulse oximetry and cardiac output estimation using volume clamp techniques [7]. Consequently, it is also likely to affect the reliability of blood pressure monitoring derived from PPG waveforms, regardless of the sophistication or quality of the ML algorithm employed.

To guarantee accurate measurements of BP with intra-arterial monitoring, one may ensure that the pressure transducer is correctly positioned at the level of the heart and the arterial pressure waveform is neither overdamped nor underdamped [17]. An overestimation of BP (mainly systolic pressure) is observed when the arterial pressure waveform is underdamped, a very common scenario in critically ill patients [18]. Arterial pressure waveforms are increasingly used to compute stroke volume and cardiac output with pulse contour algorithms [19–21]. Wrong positioning of the pressure transducer [22, 23] and damping phenomena [24, 25] may result in wrong stroke volume and cardiac output measurements and, ultimately, in wrong therapeutic decisions. Interestingly, ML algorithms have been proposed to detect improper positioning of pressure transducers and abnormal damping [26] (Table 1). In surgical patients monitored with a radial arterial catheter, Rinehart et al. [27] tested an ML algorithm trained to detect a pressure transducer that was either too low or too high and an overdamped arterial pressure waveform. They reported areas under the receiver operating characteristic curve (AUROC) ranging between 0.91 and 0.99, sensitivities between 92 and 99%, and specificities between 74 and 99% to detect all abnormal states. Of note, other methods not based on AI have also been proposed to detect and correct abnormal arterial pressure waveforms. In critically ill patients with underdamped waveforms (confirmed by the Gardner reference method), Foti et al. [25] showed that a commercially available electronic filter (MostCareUp, Vygon, France) was able to detect underdamping, normalize the BP waveform, and provide systolic BP and cardiac output values comparable to those obtained with a mechanical resonance filter. In summary, ML algorithms may help improve the quality of arterial pressure waveforms. However, they may not be indispensable to do so.

## AI for the prediction of hypotension

Proactive BP management during surgery has gained widespread interest, as intraoperative hypotension is associated with postoperative complications such as kidney failure, myocardial injury, and increased mortality [28]. The first AI model commercially available for predicting hypotension is the Hypotension Prediction Index (HPI). Based on arterial waveform features, it forecasts a mean arterial pressure (MAP) < 65 mmHg for at least one minute [29] (Table 1). A recent systematic review and meta-analysis of validation studies reported a pooled AUROC of 0.89 for predicting hypotension 5 to 15 min in advance [30].

**Fig. 2:**
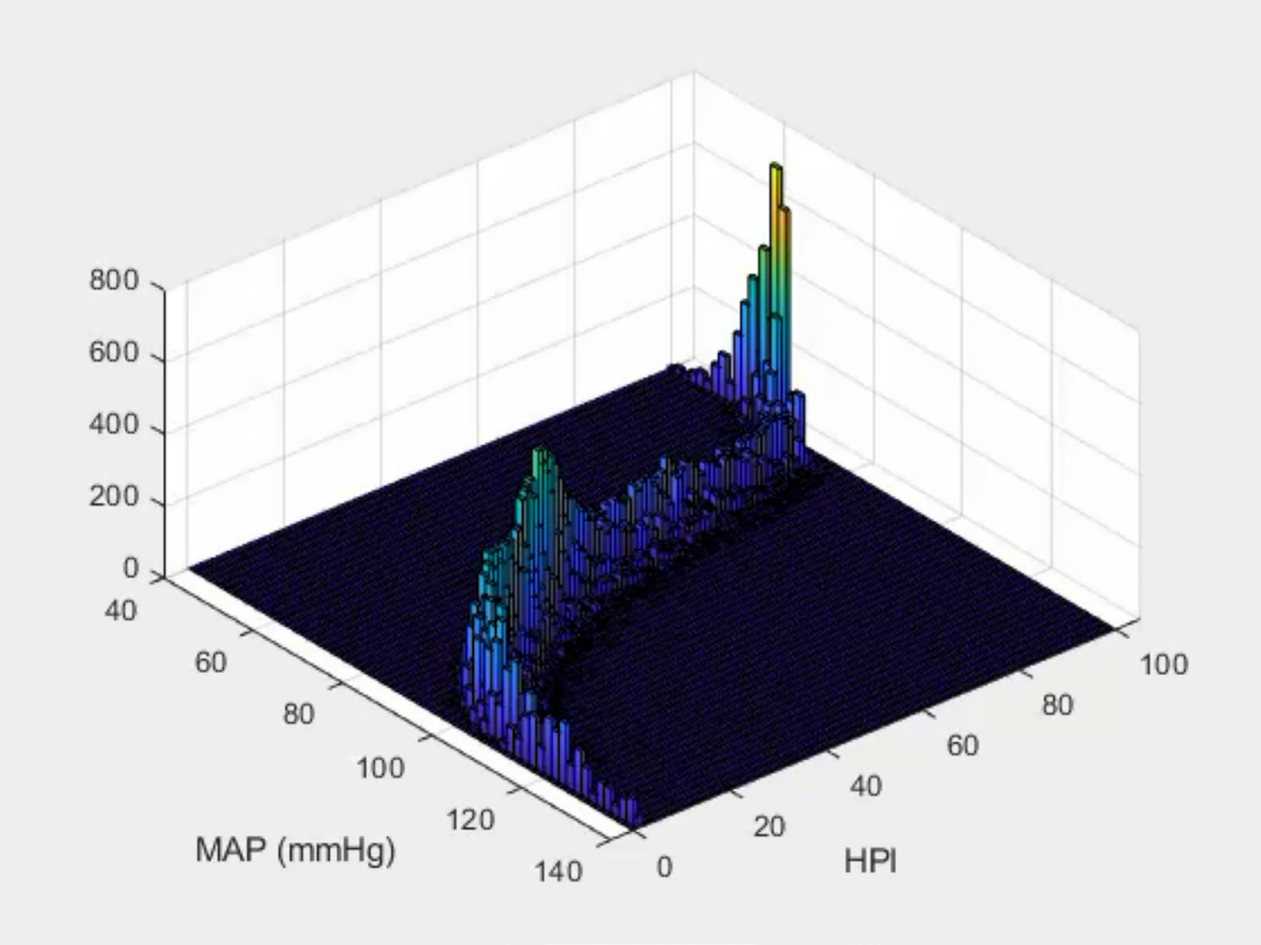
3D view of the non-linear and tight (*r* = 0.99) relationship between mean arterial pressure (MAP) and the hypotension prediction index (HPI). Original figure created with data from Mulder et al. [35]

However, concerns have emerged about the HPI development process, specifically about data leakage in the training of the model [31–33]. This indicates that information on the outcome (hypotension or non-hypotension) was used to select the input data. This is methodologically incorrect as the AI model then has pre-knowledge, and its predictive performance is artificially high. In the HPI algorithm development, the training data has been selected in such a way that the model mostly learned that the current MAP is an important predictor of hypotension. This resulted in an almost perfect symmetry between HPI and current MAP values, known as the “mirror effect” [34]. A clinical study in 100 non-cardiac surgery patients found a Spearman rank correlation of 0.99 between HPI and simultaneous MAP values (Fig. 2) [35]. Recent studies have shown that HPI is above 85 (the threshold value recommended by the developers to predict hypotension and invite clinicians to a proactive intervention) as soon as the MAP is 71–73 mmHg or below [36, 37]

**Fig. 3:**
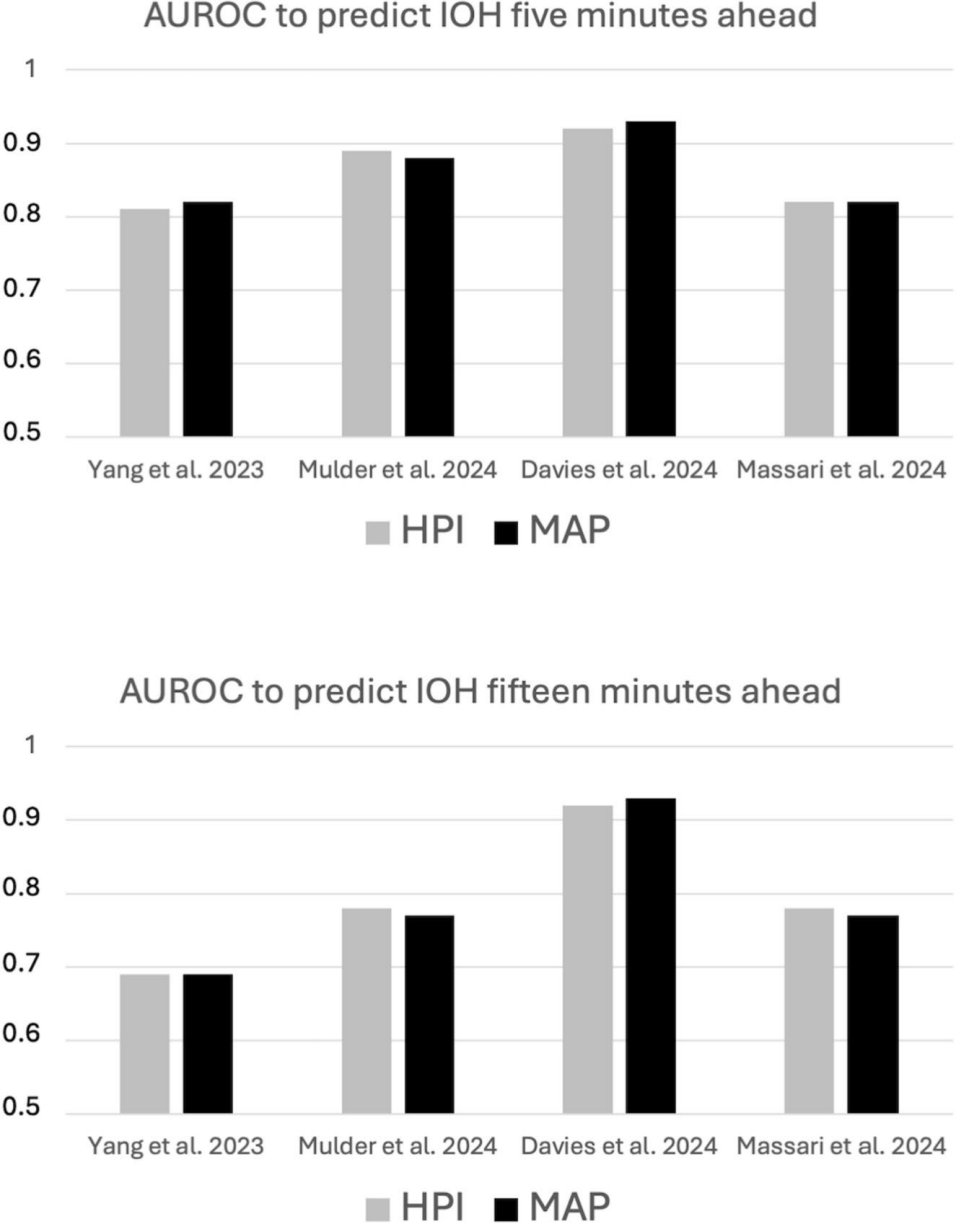
Predictive value of the Hypotension Prediction Index (HPI) and the Mean Arterial Pressure (MAP). Areas under receiver operating characteristic curves (AUROC) to predict intraoperative hypotension (IOH, defined by a MAP < 65 mmHg for at least 1 min) were compared in 4 recent clinical studies

Given the close correlation between HPI and MAP values, researchers have hypothesized that MAP alone could perform similarly in predicting future hypotension [36, 38, 39]. Recent studies have confirmed that AUROCs for HPI and MAP are virtually identical (Fig. 3) [35, 40–42]. One could imagine the AUROC to be the same for MAP and HPI, but with one variable being more specific and the other being more sensitive. This is not the case, the MAP and HPI ROC curves are perfectly superimposed [35, 41, 42], indicating that whatever the cut-off value selected to predict hypotension, the performance of both variables is comparable in terms of sensitivity and specificity.

Furthermore, the focus on AUROC as the primary performance measure, though useful for model comparison, lacks clinical relevance [43]. In practice, the positive predictive value is key to determining the proportion of false alerts that may lead to unjustified therapeutic interventions. Studies have reported low positive predictive values both for HPI and MAP. In the first truly independent HPI study (in most initial studies, data were analyzed by the developers), Ranucci et al. [44] reported a positive predictive value of only 13%. In two more recent and larger studies [35, 41], the positive predictive values were around 30%, suggesting that 7 out of 10 alarms may be false. However, the estimation of the true positive predictive value is challenging. Indeed, clinicians may react to HPI alerts by administering fluid or vasopressors, which could prevent hypotensive events. Such scenarios would be wrongly classified as false positives because HPI was high, and hypotension did not occur. Therefore, to get a fair estimation of the positive predictive value, it must be estimated from studies where HPI values are blinded. Such a study was recently published by Massari et al. [42], who reported a positive predictive value of 62%. In summary, one may reasonably estimate that around one to two-thirds of HPI alerts are false [33]. These frequent false alerts may lead to the administration of fluid, vasopressors, and/or inotropes (with known side effects) to normotensive patients who would never develop hypotension [38]. In this respect, several studies have reported increased hypertension during HPI monitoring, an observation likely due to overtreatment [45, 46]. A risk–benefit evaluation was recently published and suggested that the probability of unnecessary treatment with HPI monitoring is 10 times higher than the probability of preventing postoperative complications [33].

Despite these concerns, the HPI has been used in several randomized controlled trials that have yielded conflicting results [47, 48]. However, a meta-analysis of twelve studies found that using HPI, particularly with a predefined treatment protocol, may reduce the incidence, duration, and severity of hypotensive events [30]. Having said that, because MAP predictive performance is comparable to that of HPI, similar results might be achievable simply by setting a MAP alarm around 72 mmHg [36, 38]. It would be a more cost-effective alternative to predict impending hypotension [34–38]. Studies comparing both strategies are ongoing.

Other AI models for predicting hypotension have been explored. A systematic review found 21 studies employing ML algorithms [30]. The overall pooled AUROC for these studies was 0.79, and MAP was often the most important predictor of hypotension. Since MAP’s AUROC for predicting hypotension typically falls between 0.69 and 0.93 (Fig. 3), these ML models may also not significantly outperform conventional hemodynamic monitoring.

In summary, while AI models like the HPI show potential in predicting hypotension, their performance is closely tied to MAP, a traditional and readily available metric. A possible explanation for the inability of ML algorithms to outperform current BP monitoring could be the lack of relevant information in the training data. For example, important risk factors for developing hypotension have not been used (e.g. patient characteristics, anesthetic dose) or cannot be used (e.g. surgical manipulation or bleeding) as input for the prediction. Therefore, the clinical added value of such AI models remains unclear, especially given the risks associated with potential overtreatment [33]. Finally, more research is also needed to evaluate the impact of hypotension prevention strategies. Indeed, studies and meta-analyses suggest that targeting higher MAP values and preventing hypotension does not reduce morbidity and mortality [49–51]. In addition, the first study designed and powered (> 900 patients) to assess the outcome impact of HPI monitoring during surgery reported no reduction in acute kidney injury (the primary outcome) and no reduction in any other postoperative complications, length of stay, hospital readmission and mortality [52].

## AI for hemodynamic profiling

The concept of integrating hemodynamic variables to delineate specific hemodynamic profiles or phenotypes has long been established. Since the 1970s, critical care textbooks have included comprehensive tables that outline these profiles based on variables measured by pulmonary artery catheters. These resources have been fundamental in educating medical professionals about cardiovascular physiology, assisting in the analysis of hypotension mechanisms, and guiding therapeutic decisions. Machine learning algorithms have recently been proposed to identify hemodynamic profiles or phenotypes (Table 1).

Geri et al. [53] used hierarchical clustering with clinical and transesophageal echocardiographic data input to explore cardiovascular phenotypes in 360 patients with septic shock. Five different phenotypes were identified: patients with hyperkinetic profile (n = 84), patients with right ventricular failure (n = 81), patients with persistent hypovolemia (n = 70), patients with left ventricular systolic dysfunction (n = 64), and patients well resuscitated (n = 61). ICU mortality was the highest (50%) in patients with left ventricular systolic dysfunction and the lowest (21%) when patients were well-resuscitated. The authors acknowledged that whether their clustering approach could help clinicians optimize hemodynamic support remains to be evaluated. Guinot et al. [54] used a similar clustering approach with clinical, biological (NT-proBNP), and echo-Doppler data input to characterize various phenotypes associated with fluid overload or congestion in 145 ICU patients. Three different phenotypes emerged from the analysis: hemodynamic congestion with moderate alterations of ventricular function (n = 75), volume overload congestion with normal cardiac function (n = 50), and systemic congestion with severe alterations of biventricular function (n = 20). These phenotypes varied significantly concerning acute kidney injury and mortality rates, as well as ICU and hospital length of stay. Nevertheless, whether clinicians having access to the same information would have classified patients similarly and what might be the impact of such classification on decision-making has not been explored yet.

**Fig. 4:**
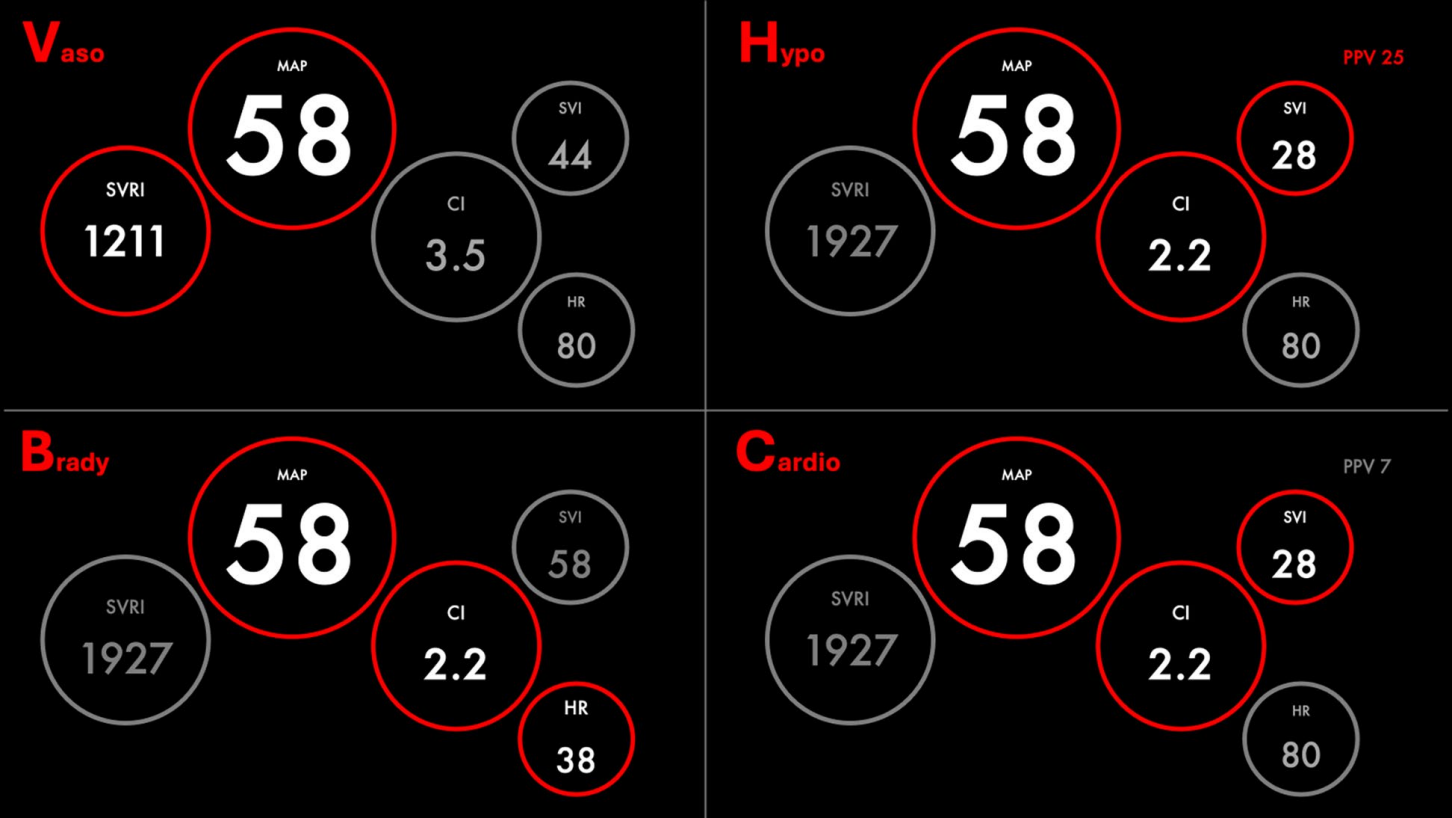
The four main hemodynamic phenotypes of hypotension. Simple visual tools (here, concept screens) enable the identification of hemodynamic profiles at a glance, without the need for AI. *Brady* bradycardia, *Cardio* Cardiac dysfunction, *CI* cardiac index, *HR* heart rate, *Hypo* hypovolemia, *MAP* mean arterial pressure, *PPV* pulse pressure variation, *SVI* stroke volume index, *SVRI* systemic vascular resistance index, *Vaso* vasodilation

In a pilot study, Kouz et al. [55] also used hierarchical clustering to identify underlying hemodynamic alterations causing intraoperative hypotension and described six different phenotypes. Based on their characteristics, the phenotypes were labeled myocardial depression, bradycardia, vasodilation with cardiac index increase, vasodilation without cardiac index increase, hypovolemia, and mixed types. In a second and larger study (2000 surgical and critically ill patients), the same group, in collaboration with industry partners, used another unsupervised deep learning model to identify four phenotypes that were labeled vasodilation, bradycardia, hypovolemia, and cardiac dysfunction [56]. The authors suggested that automation of hemodynamic profiling with AI may help reduce the cognitive load associated with integrating and interpreting hemodynamic measurements. Machine learning offers the significant advantage of rapidly processing vast amounts of data, which can be invaluable in complex clinical scenarios. However, in the context of hemodynamic monitoring, only a limited number of key variables are required to understand the mechanisms behind hypotension (e.g., vasodilation = low BP with preserved cardiac output). In addition, visual decision support tools embedded in modern hemodynamic monitors provide an accessible and effective means for clinicians to identify hemodynamic profiles at a glance. These tools simplify hemodynamic information into clear visual formats that support timely decision-making without needing the intricate computations that ML algorithms entail (Fig. 4). The necessity of integrating complex, patent-protected, and costly ML algorithms for hemodynamic profiling is, therefore, debatable. The core value of ML in this context should be carefully considered against more straightforward and cost-effective solutions that already fulfill clinical needs [57, 58]. In addition, instances were noted where patients were classified under the “bradycardia” category despite presenting with heart rates above 60 bpm, and others were marked as having “vasodilation” even when their systemic vascular resistance index was within normal or elevated ranges. These inconsistencies can have significant clinical implications. If ML algorithms are used to guide therapeutic interventions, mislabeling could lead to inappropriate treatment strategies. For example, a patient misclassified under “bradycardia” might receive chronotropic agents unnecessarily, while a patient tagged as having “vasodilation” could be inappropriately treated with vasopressors despite having normal vascular resistances. Such inaccuracies pose potential safety risks.

In summary, ML algorithms offer the potential to unlock valuable clinical insights from the extensive data recorded in critically ill and surgical patients far beyond what the human brain can process. However, they may not be essential for integrating and interpreting “small data,” such as a limited set of hemodynamic or echocardiographic variables. Evaluating ML algorithm performance necessitates comparison with established practices and simpler tools [57], like straightforward classification tables or visual decision support tools.

## AI for echocardiographic assessment

Over the last decade, ultrasound devices have become smaller, smarter, and more affordable. Most cart-based point-of-care ultrasound (POCUS) systems are light and easy to move from one room to the other. Some POCUS devices fit in the pocket, and several transducers can be connected to an electronic tablet or a smartphone, sometimes wirelessly [59, 60]. In case of hemodynamic instability, echocardiography enables a quick evaluation of cardiac anatomy and function [61, 62]. A recent systematic review and meta-analysis of 18 randomized controlled trials suggested that POCUS-guided resuscitation of shock states may reduce the duration of vasoactive medication, the need for renal replacement therapy, and 28-day mortality [63]. Echocardiography is recommended as a first-line approach for the diagnosis and early management of patients with clinical signs of circulatory shock [64, 65]. However, precise quantitative ultrasound evaluations of cardiac function may remain challenging for some clinicians, especially for trainees.

Most recent ultrasound devices come with software innovations, from speckle tracking [66, 67] to ML algorithms [68, 69], designed to facilitate and improve the quality of echocardiographic evaluations (Table 1). Machine learning algorithms have been trained using a large number of echocardiographic images and become capable of recognizing ultrasound images, guiding users to optimize image quality, automatically measuring key echocardiographic variables, and reducing the intra-operator variability of measurements [69, 70]. Most clinical validation studies published so far have been done in cardiac patients. However, several recent studies on critically ill and surgical patients yielded promising results, particularly for beginners in echocardiography (typically ICU residents).

**Fig. 5:**
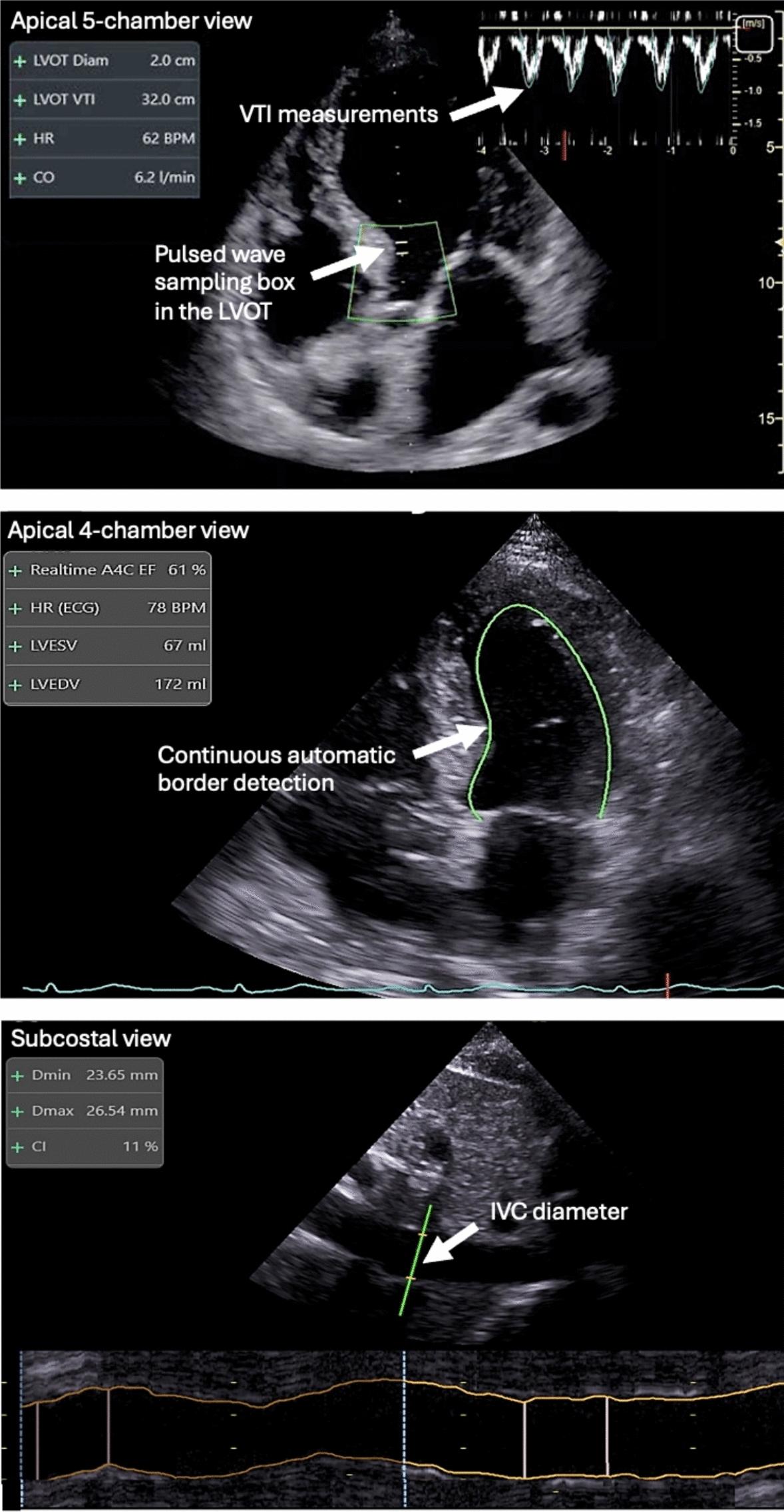
Examples of AI-enabled tools for echocardiography. Top: Automated Velocity Time Integral (VTI) measurement from an apical 5 chambers view, Middle: Automated Left Ventricular Ejection Fraction measurement from an apical 4 chambers view (Realtime A4C EF), Bottom: Automated measurement of the inferior vena cava (IVC) collapsibility index (CI) from a subcostal view. *LVOT* left ventricle outflow tract diameter, *Diam* diameter, *HR* heart rate, *CO* cardiac output, *LVESV* left ventricular end-systolic volume, *LVEDV* left ventricular end-diastolic volume, *Dmin* minimum diameter, *Dmax* maximum diameter

As of today, most ML algorithms have been developed for the real-time estimation of left ventricular ejection fraction (LVEF) [71–73]. For example, Varudo et al. [73] used a neural network algorithm that automatically detects the apical 4-chamber view, endocardial left ventricular borders, and end-diastolic/systolic times from mitral valve motion. Then, it calculates left ventricular volumes and LVEF (Fig. 5). In 95 critically ill patients, Varudo et al. [73] reported an excellent specificity (> 95%) to detect LV dysfunction. Interestingly, in this study, the reproducibility of LVEF measurements was better when novices were using the ML algorithm than when experts in echocardiography were taking the measurements manually. Other ML algorithms can directly estimate LVEF without estimating left ventricular volumes [74].

Machine learning algorithms have also been designed for the automated assessment of the subaortic velocity time integral (VTI), the respiratory variations in inferior vena cava (IVC) diameter, the mitral annular plane systolic excursion (MAPSE), and the left ventricular diastolic function. The subaortic VTI is a surrogate of left ventricular stroke volume that can be used to assess fluid responsiveness (a significant rise in VTI during a passive leg raising maneuver or a fluid challenge is observed in fluid-responsive patients) and to calculate cardiac output [75]. An ML algorithm recognizes the apical 5-chamber view and the left ventricular outflow tract (Fig. 5). Then, the pulsed wave sampling box is positioned automatically in the left ventricular outflow tract to capture the optimal Doppler signal and calculate the average VTI over a 4-s period [76]. Doing so enables a quick estimation of the sub-aortic VTI with a low percentage error, even in the hands of trainees [77]. In mechanically ventilated patients, the respiratory variation in IVC diameter has been proposed to predict fluid responsiveness [78]. In this regard, several ML algorithms have also been trained for the automated evaluation of the IVC profile, which allows the continuous tracking of the vessel diameter throughout the respiratory cycle [79]. Apart from the precise identification of the minimum and maximum IVC diameters and the automated calculation of the collapsibility or distensibility index (Fig. 5), ML algorithms have another advantage: they track the same portion of the IVC, which is somewhat impossible when using the classical M-mode sampling approach because of the IVC swing contextual to the diaphragmatic movements. Other ML algorithms have been developed for the automated calculation of the MAPSE and a quick assessment of left ventricular systolic function with transthoracic echo in critically ill patients or with transesophageal echo during surgery [80, 81]. Left ventricular diastolic dysfunction is an increasing concern for intensivists because it may dramatically impact the tolerance to fluid administration and is not detected by quick qualitative POCUS evaluations [82]. The diagnosis and the grading of diastolic dysfunction typically require the quantification of multiple echocardiographic parameters by an experienced operator, and it is time-consuming. Using ML algorithms, Chen et al. [83] developed and validated a reliable method to detect and grade left ventricular diastolic dysfunction in a few seconds, saving time and labor.

Finally, a possibly under-explored advantage of AI in echocardiography pertains to the possibility of using it for teaching. Several pathways are currently available to obtain certification in echocardiography, and an increasing number of clinicians have been trained to perform echocardiographic assessments [84]. However, human resources are limited, and the availability of trainers and supervisors may become an issue. Trainees and clinicians willing to improve their skills could benefit from the availability of AI-enabled tools to compare their manual calculations with those done automatically by the ML algorithms.

## Perspectives and challenges ahead

The rapid advancements in AI offer a glimpse into a future where ML innovations seamlessly integrate into healthcare and could be used in combination. For example, AI tools could transform the surgical patient journey, starting before hospital admission, by characterizing patient cardiovascular phenotypes and personalizing treatment pathways. In the operating room and ICU, AI tools could enhance and optimize clinical decision-making, while in surgical wards and post-discharge care, they could enable early detection of hemodynamic deterioration, preventing rescue interventions or readmissions [2, 14, 85].

Despite this promising potential, numerous challenges hinder the effective implementation of AI tools [1–3, 86, 87]. These hurdles begin at the development stage, where the availability of representative, diverse, and inclusive datasets is critical to ensuring the generalizability and equity of research outcomes. Regulatory and commercial constraints further complicate the landscape. Both regulatory bodies and developers often lack the expertise, manpower, and frameworks to evaluate the safety and clinical value of learning tools, which inherently require frequent updates.

Another pressing issue is the need for greater algorithm transparency and explainability to build trust and drive clinical adoption [86, 87]. Education plays a vital role here. Starting from medical school, clinicians should be equipped with a foundational understanding of ML principles and limitations—not to turn them into computer scientists but to demystify AI tools. This would help dispel perceptions of AI as either an omniscient "Deus ex machina" or an opaque, error-prone black box threatening their roles.

Accountability is another crucial dimension. While AI tools provide valuable insights, most clinical decisions will ultimately be made by humans. These tools are often validated in specific contexts and may not perform reliably in different scenarios. This raises significant questions about liability. Who bears responsibility in the event of a wrong diagnosis or adverse outcome—the developer or the clinician? Such concerns become even more pronounced with AI-driven closed-loop systems that not only suggest therapeutic options but also autonomously administer treatments. Predictive analytics introduce additional complexities by encouraging clinicians to act “proactively” based on probabilities rather than confirmed diagnoses [5, 38]. This represents a fundamental shift in clinical practice, as clinicians are traditionally trained to detect and treat established disease states or adverse events. Acting on probabilities, particularly when patients are hemodynamically stable and the positive predictive value is less than ideal, poses ethical and practical dilemmas—especially if the risks of harm outweigh the potential benefits [33].

The high development costs and premium pricing of most AI innovations present a significant barrier to their clinical adoption, particularly in middle-income countries. While technologies like continuous BP monitoring using a pulse oximeter or automated cardiac function assessment via a pocket ultrasound device connected to a smartphone may initially seem like practical solutions to improve access to modern hemodynamic monitoring and management, their costs may limit widespread use. Currently, the few commercially available AI solutions are sold at premium prices, restricting their accessibility to a privileged minority. For instance, the integration of the HPI into cardiac output monitoring systems has nearly doubled the cost of arterial pressure sensors. This price increase is particularly troubling given that the existing costs of cardiac output monitoring systems are already a significant barrier to their use [88]. These financial constraints reduce the adoption of such systems, thereby limiting the number of high-risk surgical or ICU patients who could benefit from advanced hemodynamic management [89].

In summary, while AI innovations hold transformative potential, they also present numerous questions and limitations that the research community and industry must address. Only by overcoming these challenges can clinicians fully embrace AI as a reliable and integral part of their daily practice.

## Conclusion

Despite significant excitement surrounding AI-enabled tools, a gap remains between the perceived potential of these technologies and their proven clinical value. Numerous studies have highlighted that ML algorithms may, at times, offer insights that clinicians can already infer or observe directly. In the realm of hemodynamic assessment for surgical and critically ill patients, certain examples stand out. For instance, the HPI has been shown to largely mirror the MAP, resulting in similar predictive capabilities for hypotensive events. Likewise, machine learning-derived hemodynamic phenotypes replicate the conventional hemodynamic profiles found in medical textbooks or shown on modern monitoring systems, albeit with some inconsistencies. These findings underscore the need for thorough validation of AI tools to ensure they provide added value beyond what is already accessible through established clinical methods.

Conversely, AI-enabled tools can offer capabilities that surpass human skills and conventional methods. Notably, they have shown the potential to track BP changes using pulse oximetry waveforms. Additionally, these tools can enable trainees to measure echocardiographic variables with greater consistency and reproducibility than even experienced clinicians performing manual assessments. Such advances hold promises for augmenting clinical precision, reducing variability, and democratizing access to high-quality patient care through supportive technology.

The creativity of engineers and computer scientists has fueled the development of exceptionally complex AI tools, which are often perceived as opaque or “black boxes” by clinicians. This makes rigorously designed clinical studies more crucial than ever. Such studies are essential for two main reasons: first, to validate the safety and effectiveness of AI-enabled tools, ensuring they perform as anticipated; and second, to confirm their superiority over existing methods in areas such as accuracy, reproducibility, speed, or accessibility. We believe that, while not every innovation will prove valuable, select AI advancements will eventually enhance care quality and patient satisfaction.

## Data Availability

Not applicable.

## Abbreviations

AI: Artificial intelligence
AUROC: Area under the receiver operating characteristic curve
BP: Blood pressure
HPI: Hypotension prediction index
IVC: Inferior vena cava
LVEF: Left ventricular ejection fraction
MAP: Mean arterial pressure
MAPSE: Mitral annular plane systolic excursion
ML: Machine learning
POCUS: Point of care ultrasound
PPG: Photoplethysmographic
VTI: Velocity time integral
